# Pathological changes and bacteriological assessments in the urinary tract of pregnant goats experimentally infected with *Brucella melitensis*

**DOI:** 10.1186/s12917-018-1533-x

**Published:** 2018-06-25

**Authors:** M. Mazlina, S. Khairani-Bejo, H. Hazilawati, T. Tiagarahan, N. N. Shaqinah, M. Zamri-Saad

**Affiliations:** 1 Research Centre for Ruminant Diseases, Faculty of Veterinary Medicine, Universiti Putra Malaysia, 43400 Serdang, Selangor Malaysia; 2 Department of Veterinary Pathology & Microbiology, Faculty of Veterinary Medicine, Universiti Putra Malaysia, 43400 Serdang, Selangor Malaysia; 3 Puncak Jalil Veterinary Clinic, Taman Puncak Jalil, 43300 Seri Kembangan, Seri Kembangan, Selangor Malaysia; 4 Department of Veterinary Laboratory Diagnosis, Faculty of Veterinary Medicine, Universiti Putra Malaysia, 43400 Serdang, Malaysia

**Keywords:** Histopathology, Immunoperoxidase, *Brucella melitensis*, Urinary tract, Goats

## Abstract

**Background:**

This study was conducted to investigate the pathological changes and distribution of *B. melitensis* in the urinary tract of pregnant goats following acute experimental infection. Six Jamnapari crossbred does in their third trimester of pregnancy were randomly assigned into two groups; Group 1 was uninfected control and Group 2 was inoculated conjunctival with 0.1 mL of the inoculums containing 10^9^ cfu/mL of live *B. melitensis.* All does were sacrificed 30 days post-inoculation before the kidney, ureter, urinary bladder, urethra and vaginal swab were collected for isolation of *B. melitensis*. The same tissue samples were fixed in 10% neutral buffered formalin for hematoxylin and eosin, and immunoperoxidase staining.

**Results:**

None of the goats showed clinical signs or gross lesions. The most consistent histopathology finding was the infiltration of mononuclear cells, chiefly the macrophages with few lymphocytes and occasionally neutrophils in all organs along the urinary tract of the infected goats of Group 2. Other histopathology findings included mild necrosis of the epithelial cells of the renal tubules, congestion and occasional haemorrhages in the various tissues. Kidneys showed the most severe lesions. Immunoperoxidase staining revealed the presence of *B. melitensis* within the infiltrating macrophages and the epithelium of renal tubules, ureter, urethra and urinary bladder. Most extensive distribution was observed in the urinary bladder. *Brucella melitensis* was successfully isolated at low concentration (3.4 × 10^3^ cfu/g) in the various organs of the urinary tract and at high concentration (2.4 × 10^8^ cfu/mL) in the vaginal swabs of all infected goats. Although *B. melitensis* was successfully isolated from the various organs of the urinary tract, it was not isolated from the urine samples that were collected from the urinary bladder at necropsy.

**Conclusion:**

This study demonstrates the presence of low concentrations of *B. melitensis* in the organs of urinary tract of pregnant does, resulting in mild histopathology lesions. However, *B. melitensis* was not isolated from the urine that was collected from the urinary bladder.

## Background

Brucellosis is a zoonotic disease that causes chronic debilitating disease in humans and major economic losses to livestock farmers [1, 2]. Caprine brucellosis is caused by *Brucella melitensis,* which has been recognized as the most pathogenic species of the Genus *Brucella.* It is least host-specific and is associated with most cases of human brucellosis [3]. As an intracellular parasite, the bacterium is able to manipulate the host’s immune system and flourishes within the professional and non-professional phagocytic cells. Thus, it can replicate within these cells since it is protected from the humoral antibodies and antibiotic treatments [4, 5].

In some cases, infected goats and sheep appear healthy with no apparent clinical sign but usually become lifelong carriers that disseminate the disease [6]. Accurate detection followed by successful removal of carriers and infected animals are imperative to reduce the cases of brucellosis [6–8].

Brucellosis in animals has always been associated with the disorders of the reproductive and reticulo-endothelial systems [5] causing abortion and enlarged spleen and liver [1, 9, 10] and less frequently affecting the musculo-nervous systems [10, 11]. *Brucella*-induced urinary tract infection is considered extremely rare in animals and humans [12]. To the best of our knowledge, no comprehensive study was done to assess the lesions in the urinary system of female goats following infection with *B. melitensis*. Thus, this study was aimed at determining the distribution of *B. melitensis* and the associated lesions in the urinary tract of does following acute experimental infection with *B. melitensis* in the third trimester of pregnancy.

## Methods

### Bacterial inoculums

A local strain of *B. melitensis* that was isolated from an outbreak of caprine brucellosis in Malaysia was used in this study [13]. The isolate was cultured onto *Brucella* Agar (BBL™, UK) for 4 days at 37 °C and later transferred into *Brucella* broth (BBL™, UK) and was further incubated at 37 °C for another 4 days in an orbital shaker incubator (YIH-DER LM-510, Taiwan). The bacterial cells were then harvested following a series of washing with sterile PBS (pH 7.4) and centrifugation at 5,000×*g*, 4 °C for 10 min each cycle. The final pellet was then diluted in sterile PBS to a final bacterial concentration of 10^9^ cells/mL using McFarland’s Standard.

### Preparation of hyperimmune serum against *Brucella melitensis*

The local strain of *B. melitensis* was grown in 35 mL of *Brucella* broth (BBL™, UK) in shaking incubator for 4 days at 37 °C. The bacterial concentration in the broth was determined using the standard total plate count method. The cells were re-suspended in sterile PBS to obtain a final concentration of 1 × 10^9^ cfu/mL, were then killed by adding 0.5% formalin and were emulsified with Freund’s complete adjuvant (FCA) (Sigma-Aldrich, US) at 1:1 ratio. One mL of the emulsion was injected subcutaneously into rabbits. Booster doses of the emulsified inoculums, prepared using Freund’s incomplete adjuvant (FIA) (Sigma-Aldrich, US) were injected on days 14 and 21. Finally, the hyperimmune serum was harvested at 28 days post-inoculation. The Institutional Animal Care and Use Committee (IACUC) of Universiti Putra Malaysia approved this protocol (AUP No: R019/2014).

### Animals and management

A total of 6 clinically healthy Jamnapari crossbred does of about 3–4 months pregnant were obtained from a farm with no history of brucellosis. They were subjected to the Rose Bengal Plate test (RBPT) and Complement Fixation test (CFT) to ensure the brucellosis-free status. The goats were then divided equally into 2 groups; the uninfected control (Group 1) and the infected (Group 2) groups. Does of Group 1 were exposed to 100 μL of sterile PBS via the conjunctiva sac. On the other hand, does of Group 2 were similarly exposed to 100 μL of the inoculums containing 10^9^ cfu/mL of live *B. melitensis*. The infected does were kept entirely in a restricted and isolated housing facility while the control goats were kept separately in a raised house with slatted floor. All does were fed with Napier grass and supplemented with palm kernel cake at the rate of 400 g/animal/day while drinking water was available ad libitum.

Following the infection, the does were monitored twice daily for clinical signs especially abortion before they were euthanized 30 days post-inoculation. All euthanasia were carried out in an isolated area at a government slaughter house using the standard electrical stunning and exsanguinations protocol, where animals were unconscious following the stunning prior to exsanguination. Post-mortem was carried out immediately and the various sections of the urinary tract encompassing the kidneys, ureter, urinary bladder, urethra, urine and vaginal swabs were collected for bacterial isolation and identification, and for histopathology examination and immunoperoxidase staining. At the same time, urine samples were collected from the urinary bladder into a sterile bottle for bacteriological isolation. For comparison, the uterus and vaginal swabs were also collected and subjected to the same process. The Institutional Animal Care and Use Committee (IACUC) of Universiti Putra Malaysia approved this study (AUP No: R019/2014).

### Histopathology

The formalin-fixed samples were processed in tissue processor (Leica TP 1020, Germany) and Tissue Embedding Console System (Leica EG1150) before they were embedded in paraffin wax and sectioned using the rotary microtome (Leica Jung Multicut 2045, Germany) at 4 μm thick. The mounted tissue sections were stained with Harris’ haematoxylin and eosin (HE). The slides were viewed under light microscope (Nikon Eclipse 50i, Japan) installed with Nikon imaging software (NIS-Elements D 3.2, Japan). The histological changes were noted and scored as 0: none, 1: 30% affected, 2: 30–60% affected and 3: > more than 60% affected [3]. All evaluations were duplicated and 5 microscopic fields of each slide were randomly selected for lesion scoring. The scores were reported as the average value of each lesion and average value for overall scoring.

### Immunoperoxidase staining

The formalin-fixed, paraffin-embedded sections were fixed on Poly-L-Lysine (Sigma- Aldrich, USA) coated microscope slides. Then they were subjected to deparaffinization and rehydration before antigen retrieval was done in citrate buffer for 10 min. Indigenous peroxidase was inactivated using 3% hydrogen peroxide followed by protein block with 5% bovine serum albumin for 15 min. Rabbit hyperimmune serum was used as the primary antibody at 1:100 dilution, incubated overnight at 4 °C. Then, secondary antibody, the goat anti-rabbit IgG (Abnova, Taiwan) diluted to 1:500 was poured and incubated for 30 min at 37 °C. All slides were developed with DAB Chromogen (Dako, USA) and counterstained with Harris’ haematoxylin, dehydrated and mounted. Control sections used normal rabbit serum as primary antibody. The slides were viewed under light microscope (Nikon Eclipse 50i, Japan), which was installed with Nikon imaging software (NIS-Elements D 3.2, Japan). The presence and distribution of IP staining were scored as 0: none, 1: focal, 2: multifocal and 3: diffuse [14]. All evaluations were done in duplicate and 5 microscopic fields were randomly selected for lesion scoring. The scores were recorded as average value of each distribution and intensity.

### Bacterial isolation from tissues

The tissue samples were flamed and then placed into sterile zipper plastic bags to minimize contamination. Then, sterile PBS (pH 7.4) was added into the zipper bag at tissue to PBS ratio of 1:2. The samples were then pounded using mortar and pestle. The resultant mixture was used for bacterial culture and extraction of bacterial DNA. The urine samples (4–5 mL) were collected using needle and syringe into a sterile tube and immediately transported to the laboratory. About 10 μL of the tissue mixture and urine sample was cultured onto *Brucella* agar that was pre-added with *Brucella* Selective Supplement (Oxoid, England) and incubated at 37 °C for 10 days. Bacterial colonies that appeared small, rounded, smooth and translucent, glistening and bluish were highly suggestive of *B. melitensis* [11] and were confirmed using PCR. The results were presented as percentage (%) of positive samples over total number of samples.

### Bacterial DNA extraction

The bacterial DNA was extracted according to the manufacturer’s recommendations (NucleoSpin® Tissue DNA Purification Kit, Macherey-Nagel, German). The extraction was initiated by adding 75 μL of the processed tissue with 25 μL of Proteinase K solution and 180 μL of lysis buffer (Buffer T1) followed by rigorous vortex before incubation at 56 °C for 3 h to lyse the samples. The mixture was vortexed regularly during the incubation period. Then, 200 μL of Buffer B3 (Lysis buffer) was added, vortexed and incubated at 70 °C for 10 min. This was followed by adding 210 μL of absolute ethanol into the mixture and vortexed. The solution was transferred into tissue columns in collecting tubes and centrifuged for 1 min at 11,000 x *g*. The flow-through was discarded before the silica gel within the tissue column was washed twice; first by adding 500 μL of Buffer BW followed by centrifugation and later 600 μL of Buffer B5. Then, the mixture was centrifuged at 11,000×*g* for 2 min to remove residual ethanol. The tissue columns were transferred into 1.5 mL micro-centrifuge tubes and 100 μL of pre-warmed 70 °C of Buffer BE was added and left at room temperature for 1 min. The elution containing highly pure DNA was obtained following centrifugation at 11,000×*g* for 1 min. The DNA was stored at -20 °C until used.

### Polymerase chain reaction

The bacterial colonies and DNA extracts were used as templates for confirmation of *B. melitensis* using the forward P1 (5’-CATGCGCTATGTCTGGTTAC-3′) and P2 (5’-AGTGTTTCGGCTCAGAATAATC-3′) primer sequences that amplified the fragment at 252 bp [15]. The PCR was performed in 25 μL reaction mixture that contained 2.5 μL of 10× buffer, 3 mM MgCl2, 400 μM dNTPs, 500 nM of each primer, 1.5 U Taq polymerase (MBI Fermentas, Lithuania) and 1 μL of purified DNA or bacterial colony mixed with 1 μL of DNAzol® reagent (Thermo Fisher Scientific, USA). The PCR amplifications were performed in a Master Cycler Pro S (Eppendorf, Germany) in 34 cycles with an initial denaturation at 95 °C for 2 min and denaturation step for 1.15 min at 95 °C. The annealing, extension and final extension phases were set at 57.1 °C for 2 min, 72 °C for 2 min and 73 °C for 5 min, respectively. The PCR products were mixed with 1 μL of loading dye and were electrophoresed through 1% (*w*/*v*) agarose gel pre-mixed with RedSafeTM Nucleic Acid Staining solution (INTRON, Korea) in 1× TBE at 80 V (Bio-Rad PowerPacTM Basic, USA) for 45 min. Five μL of 100 bp DNA marker (GeneDireX®, Taiwan) was run simultaneously. The bands were documented using the gel documentation software called GeneSnap®, UK.

## Results

### Histopathology changes and IP staining

**Fig. 1:**
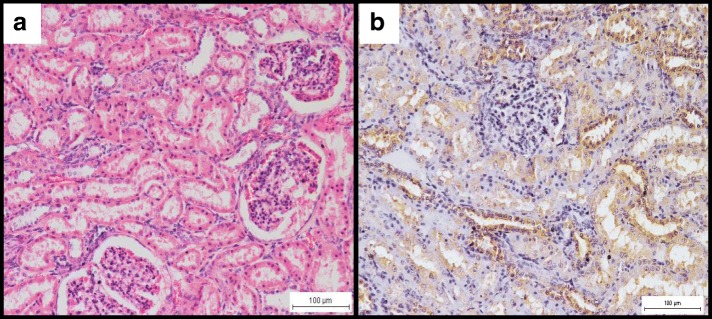
**a** Moderate congestion of the glomerulus and mild glomerulonephritis with infiltration of mainly neutrophilic cells. H&E, × 200. **b** Diffuse and moderately strong immunoperoxidase stain reactions in the epithelial cells of the renal tubules and in the cytoplasm of inflammatory cells infiltrating the glomeruli. IHC, × 200

Infected does displayed mild glomerulonephritis with neutrophils, glomerular congestion and mild renal tubular necrosis (Fig. 1a). Haemorrhages and foci of inflammatory reactions were occasionally observed in the interstitium. Immunoperoxidase staining of the kidneys revealed strong to moderate staining (Fig. 1b)*.* Strong reactions were observed in the cytoplasm of the inflammatory cells infiltrating the glomeruli and renal tubules, particularly the distal convoluted and the collecting tubules. Milder immunoreactions were noted in the epithelial cells of the proximal convoluted tubules and occasionally in the lumen of renal tubules. *Brucella melitensis* was isolated from 3 of the 6 (50%) kidney samples at an average rate of 3.9 × 10^3^ cfu/g of tissue compared to 4 out of 6 (66.7%) kidney samples that were positive PCR (Table 1).

**Table 1:** Rate of isolation and average concentration of *Brucella melitensis* in the various organs of the urinary tract of pregnant goats following acute infection

Group/Organs	Kidneys	Ureter	Urinary Bladder	Urethra	Average
G1	0/6 (0%)	0/6 (0%)	0/3 (0%)	0/3 (0%)	0%
G2	3/6 (50%)	3/6 (50%)	1/3 (33%)	2/3 (67%)	50%
	[3.9 × 10^3^] ^a^	2.9 × 10^3^] ^a^	[4.2 × 10^3^] ^a^	[2.6 × 10^3^] ^a^	[3.4 × 10^3^] ^a^

^a^unit = cfu/g tissue

**Fig. 2:**
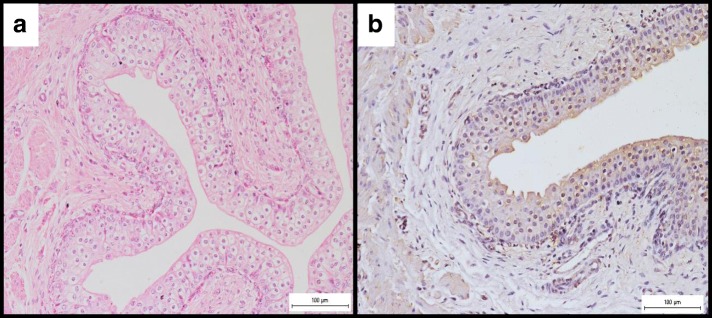
**a** Mild congestion and ureteritis characterized by infiltration of macrophages in the connective tissue stroma. H&E, × 200. **b** Positive immunoperoxidase stain reactions in the cytoplasm of the epithelial cells of the ureter and within the infiltrating macrophages. IHC, × 200

There was also mild inflammation in the ureter (Fig. 2a) with macrophages in the lamina propria and connective tissue stroma. The immunoperoxidase revealed positive staining, particularly within the macrophages and the epithelial cells (Fig. 2b). Again, *B. melitensis* was successfully isolated from 3 out of 6 (50%) of the ureter samples at the rate of 2.9 × 10^3^ cfu/g of tissue compared to 4 out of 6 (66.7%) of the ureter were positive PCR.

**Fig. 3:**
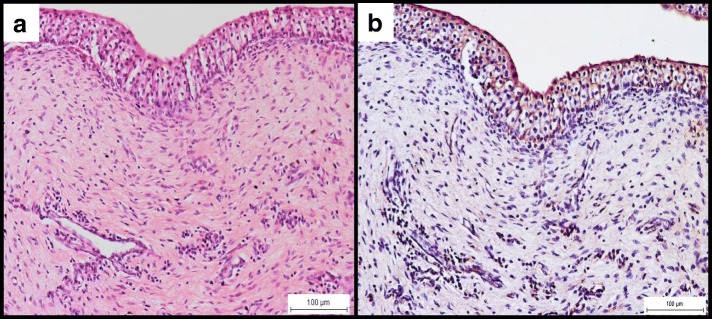
**a** Cystitis with macrophages and few lymphocytes in the connective tissue layer of the urinary bladder. H&E, × 200. **b** Strong golden brown immunoperoxidase staining observed intracellularly in macrophages and in the transitional epithelial cells of the urinary bladder. IP, × 200

**Fig. 4:**
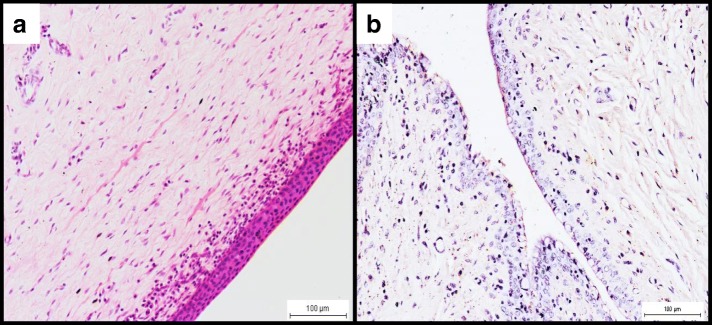
**a** Infiltration of few macrophages at the perivascular area and the submucosa of urethra. H&E, × 100. **b** Positive light golden brown immunoperoxidase staining observed in the cytoplasm of the macrophages and epithelial cells of the urethra. IP, × 100

All infected does had cystitis, characterised by intense perivascular inflammation with predominantly macrophages (Fig. 3a). The immunoperoxidase staining revealed immunoreactions within the transitional epithelial cells as well as in the cytoplasm of the macrophages (Fig. 3b). The bacterium was isolated from 1 out of 3 (33.3%) bladder samples at the rate of 4.2 × 10^3^ cfu/g of tissue while PCR was 66.7% (Table 1). Similarly, the urethra of all infected does showed massive infiltration of mainly macrophages at the submucosal and perivascular regions (Fig. 4a). The immunoperoxidase stained the transitional epithelial cells and the cytoplasm of macrophages (Fig. 4b). Attempts to isolate *B. melitensis* from the urethra yielded 2 out of 3 (66.7%) at an average rate of 2.6 × 10^3^ cfu/g of tissue while PCR revealed 100% positive results. In comparison, *B. melitensis* was isolated from 2 out of 3 (66.7%) vaginal swab samples at a higher concentration of 2.4 × 10^8^ cfu/mL but isolation was unsuccessful from any of the urine sample. The control uninfected goats revealed generally normal histological features with no inflammatory reaction and positive IP staining while *B. melitensis* was not isolated from any organ.

### Lesions and immunoperoxidase scorings

None of the infected pregnant does of Group 2 exhibited clinical signs following experimental infection. However, 4 kids were eventually delivered weak by the infected does and none survived the first month after birth. Necropsy revealed no obvious gross lesions in the urinary system. Nevertheless, histopathological examinations revealed notable microscopic changes and immunoperoxidase staining in various sections of the urinary tract of the infected goats of Group 2 (Table 2). The average lesion score for kidney was significantly (*p* < 0.05) higher (score 0.61 ± 0.28) than the urinary bladder (score 0.31 ± 0.15). However, the immunoperoxidase staining was significantly (*p* < 0.05) higher in the urinary bladder (score 2.01 ± 0.07) than the kidneys (score 1.33 ± 0.13) and gradually but significantly (*p* < 0.05) decreasing from urinary bladder to the urethra. The uterus of the infected goats showed significantly (*p* < 0.05) most severe histological lesions but IP distribution score was similar (*p* > 0.05) to those of urinary bladder (Table 2). None of the control uninfected goats of Group 1 showed clinical signs, gross and histopathological changes, and immunoperoxidase staining.

**Table 2:** Average histopathology and immunoperoxidase scores (mean ± SE) in the various ogans of the urinary tract of pregnant goats following acute experimental infection with *B. melitensis*

Organ	Histology Score	IP Score
	(Mean ± SE)	(Mean ± SE)
Kidney	0.61 ± 0.28^a^	1.33 ± 0.13^a^
Ureter	0.21 ± 0.15^b^	0.47 ± 0.18^c^
Urinary bladder	0.31 ± 0.15^b^	2.00 ± 0.07^b^
Urethra	0.21 ± 0.68^b^	0.13 ± 0.07^d^
Uterus	0.84 ± 0.33^c^	1.80 ± 0.42^c^

^a,b,c,d^Different superscripts represent significant (*p* < 0.05) difference of data within the respective column

## Discussion

There is a lack of information describing the pathological lesions and distribution of *B. melitensis* in the urinary tract of goats. This study revealed that the most common infiltrating inflammatory cell in the organs of urinary tract is the macrophage [16] while *B. melitensis* was present mostly within the cytoplasm of these macrophages. These are in agreement with earlier studies involving other organs, particularly organs of the reproductive tract [3, 14, 17]. Studies on *B. abortus* have reported its ability to reside and replicate in the macrophages [18] while hiding from the humoral immune responses and the effects of antibiotic treatments [5]. More importantly, the bacterium manipulates the macrophages by turning them into reluctant factory for massive replication of *Brucella* [19]. Apart from that, macrophages are unwilling agents of dispersal, responsible for the widespread dissemination of the bacterium [3, 20]. Furthermore, this study has proven that *B. melitensis* can invade non-phagocytic cells such as the epithelial and fibroblast cells [18, 21].

*Brucella melitensis* has been detected in the kidneys of West African Dwarf goats where the antigen was found mostly in the epithelium of renal tubules and glomeruli [14]. Similarly, *B. ovis* was successfully cultured from the kidneys and the urinary bladder of infected stags [15]. However, both reports did not mention the presence of *Brucella* in the urine. Furthermore, the concentrations of *B. melitensis* isolated from the various organs of the urinary tract in this study were relatively low compared to the reproductive tract. This might be the reason for the absence of *B. melitensis* in urine. Furthermore, immunoperoxidase staining revealed positive staining within the macrophages and relatively mild stain in the urinary epithelial cells. Therefore, the low level of *B. melitensis* within the epithelial cells of urinary tract was not enough to be excreted into the urine although excretion of *Brucella* in urine of infected animals has been occasionally reported [22]. Similar involvement of human urinary tract in brucellosis has been reported but the involvement is considered rare and almost always occur together with the reproductive system [12, 23].

Immunoperoxidase staining is a tool for detection of *B. melitensis* in tissues. It is highly specific and capable of showing the distribution of *Brucella* in affected tissues [3]. Using immunoperoxidase, *B. melitensis* has been shown to have tropism for the macrophages and the epithelial cells of urinary tract. Furthermore, polymerase chain reaction (PCR) has been used lately for antigen detection in infected tissues with high specificity and sensitivity [15, 22, 24]. Nevertheless, bacterial isolation is still the irrefutable method to confirm the presence of the pathogen thus, considered the gold standard for diagnosis of brucellosis [10, 24, 25]. Our attempts to isolate *B. melitensis* from the various organs of the urinary tracts were successful while PCR is deemed useful in detecting nucleic acid fragments of the bacterium [15, 22, 24, 25].

## Conclusions

In conclusion, acute infection of pregnant goats by *B. melitensis* in this study led to mild lesions in the organs of the urinary tract. Low concentrations of *B. melitensis* were found within the macrophage and epithelial cells. However, it was not isolated from the urine samples that were collected from infected urinary bladder.

## Abbreviations

CFT: Complement fixation test
cfu/g: Colony-forming unit per gram
cfu/L: Colony-forming unit per litre
DAB: 3.3′-Diaminobenzidine
DNA: Deoxyribonucleic acid
FCA: Freund’s complete adjuvant
FIA: Freund’s incomplete adjuvant
HE: Haematoxylin and eosin
IACUC: Institutional Animal Care and Use Committee
IgG: Immunoglobulin G
IP: Immunoperoxidase
PBS: Phosphate buffered saline
PCR: Polymerase chain reaction
RBPT: Rose Bengal Plate Test
